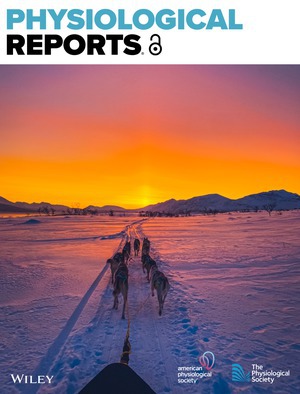# Cover Image

**DOI:** 10.14814/phy2.70753

**Published:** 2026-01-29

**Authors:** Silje Sælen‐Helgesson, Anne Dragøy Hafstad, Trine Lund, Ingebjørg Helena Nymo, Chiara Ciccone, Shona Hiedi Wood, Lars P. Folkow, Monica Alterskjær Sundset

## Abstract

The cover image is based on the article Effects of endurance training on skeletal muscle mitochondrial respiration in Siberian and Alaskan huskies by Monica Sundset et al., https://doi.org/10.14814/phy2.70725